# Paraneoplastic Leukemoid Reaction in Lung Adenocarcinoma With Multi-lineage Granulocytic Abnormalities and Eosinophilic Pleural Effusion

**DOI:** 10.7759/cureus.97404

**Published:** 2025-11-21

**Authors:** Yongqiu Duan, Yang Yang, Hongwei Yu, Chunxiu Zhang, Yiting Wang, Min Ruan, Zeng Yunyan

**Affiliations:** 1 Department of Respiratory and Critical Care Medicine, The Second People's Hospital of Baoshan, Baoshan, CHN

**Keywords:** eosinophilic pleural effusion, lung adenocarcinoma, multi-lineage granulocytic proliferation, paraneoplastic leukemoid reaction, rapid progression

## Abstract

This report describes a rare and lethal phenotype of paraneoplastic leukemoid reaction (PLR) in lung adenocarcinoma, defined by a triad of multi-lineage myeloid proliferation, marked eosinophilic malignant pleural effusion, and progressive lymphopenia. The patient presented with a hyper-aggressive clinical course, culminating in a fatal outcome within 12 days, suggesting an underlying multi-cytokine storm. Clinically, this triad serves as a critical alert for distinguishing PLR from infection and underscores the importance of early bone marrow evaluation for rapid diagnosis. This case highlights the urgent need for mechanistic studies of multi-cytokine-driven PLR.

## Introduction

Paraneoplastic leukemoid reaction (PLR) is a rare hematologic complication of malignancy associated with a grave prognosis. While the incidence of PLR in lung cancer is less than 1% [1], it typically manifests as a neutrophil-predominant pattern [2-4]. Although occasional cases with eosinophilia have been described [5-7], a comprehensive report of lung adenocarcinoma-associated PLR featuring the concurrent triad of multi-lineage granulocytic proliferation, marked eosinophilic malignant pleural effusion, and progressive lymphopenia has not been previously documented. We report this educational case to delineate this unique, hyper-acute clinical course and to improve its recognition, diagnosis, and prognostic assessment.

## Case presentation

A 77-year-old man was admitted with an eight-day history of cough and chest tightness, which had not improved with antibiotics and tube thoracostomy at an outside hospital. On admission, empirical treatment with cefoperazone-sulbactam, which provides broad-spectrum coverage including for common Gram-positive organisms, was initiated.

**Figure 1:**
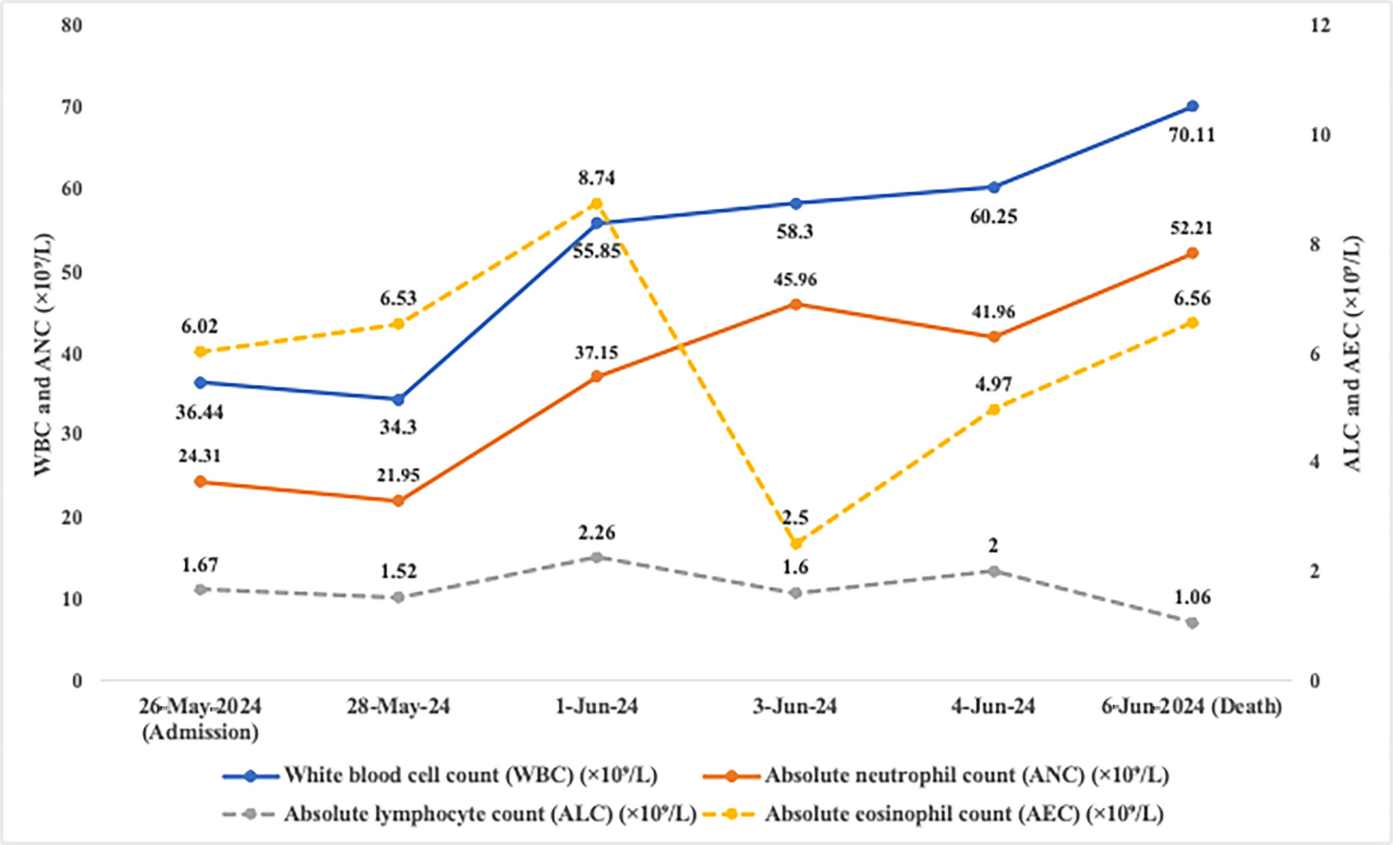
Dynamic changes in key hematologic parameters The line graph visually represents the data from Table 1. It illustrates the rapid and progressive increase in white blood cell (WBC) and absolute neutrophil counts (ANC) on the primary Y-axis (left). In stark contrast, the absolute lymphocyte count (ALC) shows a declining trend, while the absolute eosinophil count (AEC) exhibits significant fluctuation, both plotted on the secondary Y-axis (right). These divergent trends visually underscore the patient's hyper-inflammatory state and concurrent immune depletion.

The patient's hematologic parameters showed a progressive and dramatic derangement (Table 1 and Figure 1). His white blood cell count escalated from 36.44×10⁹/L on admission to a peak of 70.11×10⁹/L by day 12, driven by a synchronous proliferation across multiple granulocytic lineages, including neutrophils, eosinophils, and basophils. In stark contrast, a progressive depletion of lymphocytes was observed. Laboratory findings were notable for a markedly elevated C-reactive protein (CRP) level (>130 mg/L) with only a minimal increase in procalcitonin (PCT) (<0.60 ng/mL).

**Table 1:** Dynamic changes in hematologic parameters and inflammatory biomarkers WBC: white blood cell count; ANC: absolute neutrophil count; AEC: absolute eosinophil count; AMC: absolute monocyte count; ABC: absolute basophil count; CRP: C-reactive protein; PCT: procalcitonin; ALC: absolute lymphocyte count

Blood	Date						Reference range
	26-May-2024 (admission)	28-May-24	1-Jun-24	3-Jun-24	4-Jun-24	6-Jun-2024 (death)	
WBC	36.44	34.3	55.85	58.3	60.25	70.11	3.2-9.5×10^9^/L
ANC	24.31	21.95	37.15	45.96	41.96	52.21	1.80-6.30×10^9^/L
ALC	1.67	1.52	2.26	1.60	2.00	1.06	1.10-3.20×10^9^/L
AMC	3.26	3.26	4.24	4.88	4.54	5.06	0.10-0.60×10^9^/L
AEC	6.02	6.53	8.74	2.50	4.97	6.56	0.02-0.52×10^9^/L
ABC	0.19	0.20	0.41	0.08	0.07	0.11	0.00-0.06×10^9^/L
Lymphocyte	4.60	4.40	4.00	2.70	3.30	1.50	20.00-50.00%
Neutrophil	66.8	64.10	66.60	78.90	69.80	74.40	40.00-75.00%
Eosinophil	16.50	19.00	15.60	4.30	8.20	9.30	0.40-8.00%
Immature granulocyte	2.70	2.40	5.50	5.60	11.10	5.16	0.00-0.60%
Absolute immature granulocyte count	0.99	0.84	3.05	3.28	6.71	7.40	0.00-0.60×10^9^/L
CRP	N/A	135.7	103	N/A	N/A	159	0.0-6.0 mg/L
PCT	0.39	N/A	0.56	N/A	N/A	1.56	<0.05 ng/mL

**Figure 2:**
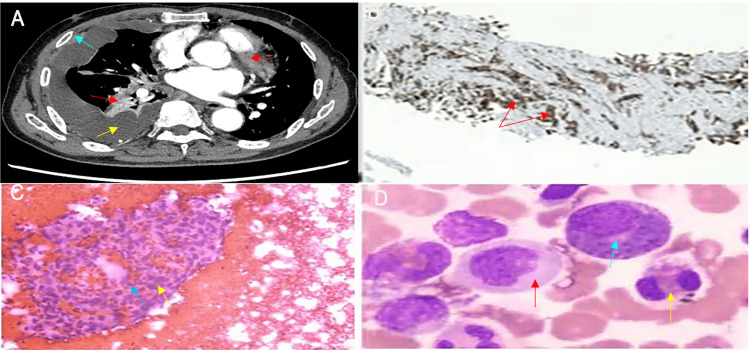
Radiologic, histopathologic, cytologic, and bone marrow findings (A) Contrast-enhanced chest CT demonstrates a large mass in the right hilar region (red arrow), accompanied by a large right-sided malignant pleural effusion (yellow arrow) and significant pleural thickening (blue arrow). (B) Immunohistochemical staining of the pleural biopsy specimen reveals strong nuclear positivity for TTF-1 in tumor cells (brown staining, red arrows), confirming an origin of lung adenocarcinoma. (C) Pleural effusion cytology (H&E stain) reveals clusters of adenocarcinoma cells against a hemorrhagic background, exhibiting enlarged, hyperchromatic nuclei and marked atypia (blue and yellow arrows). (D) Bone marrow smear demonstrates marked granulocytic hyperplasia. Myeloblasts (red arrow), plasma cells (blue arrow), and mature eosinophils (yellow arrow) are visible, features consistent with a paraneoplastic leukemoid reaction. CT: computed tomography; TTF-1: thyroid transcription factor-1; H&E: hematoxylin and eosin

A contrast-enhanced chest computed tomography (CT) scan confirmed an extensive tumor burden in the right lung and pleura (Figure 2A). A pivotal finding was obtained from pleural fluid analysis, which revealed a hemorrhagic exudate with a high white blood cell count and an eosinophil fraction of 30.7% (Figure 2C). Critically, Gram stain, bacterial cultures, and fungal cultures of both the pleural fluid and blood samples were all negative. After excluding parasitic infections and allergic diseases, the eosinophilic effusion was attributed to a tumor-associated mechanism. The diagnosis of stage IV lung adenocarcinoma with bone marrow metastasis was established by pleural and bone marrow biopsies (Figure 2B, Figure 2D). The bone marrow aspirate was consistent with a leukemoid reaction.

The final diagnosis was stage IV lung adenocarcinoma with pleural and bone marrow metastases, complicated by PLR. The patient's clinical condition deteriorated rapidly. Despite broadening antibiotic coverage to meropenem for a fever that developed on hospital day 9, his leukocytosis and inflammatory markers continued to rise. This refractory pattern, combined with the negative culture results, strongly indicated a cytokine-driven paraneoplastic response rather than an infectious process. The patient died of respiratory and circulatory failure on hospital day 12, precluding the initiation of any antineoplastic therapy. Due to the patient's rapid clinical deterioration, comprehensive next-generation sequencing was not performed.

## Discussion

This report describes an exceptionally rare and aggressive subtype of PLR in a patient with lung adenocarcinoma. In contrast to the classical granulocyte colony-stimulating factor (G-CSF)-mediated, neutrophil-predominant pattern [2,4], this case was defined by three key features: multi-lineage myeloid proliferation, a marked eosinophilic malignant pleural effusion, and progressive lymphocyte depletion. This clinical triad broadens the known phenotypic spectrum of PLR and highlights potential diagnostic pitfalls.

Diagnostic challenges: PLR versus infection

The clinical presentation of our patient closely mimicked severe sepsis. The CRP level was markedly elevated, while PCT was only mildly increased, and the patient showed no response to broad-spectrum antibiotics, including cefoperazone-sulbactam and meropenem. Without a thorough evaluation of serial hematologic trends (such as a sustained fall in the lymphocyte percentage), pleural fluid cytology, and bone marrow examination, this clinical picture could easily be misclassified as a refractory infection, leading to a delay in diagnosis. Therefore, the combination of a disproportionate CRP-to-PCT elevation, unresponsiveness to antibiotics, and extreme leukocyte derangements should serve as a critical warning sign for PLR.

Furthermore, a persistent decline in the lymphocyte percentage is a key indicator of immune exhaustion and a grave prognosis, which should not be overlooked even if the absolute count remains within the reference range. A significant eosinophil fraction (>10%) in the pleural fluid should also be considered a high-risk marker [6,8], potentially suggesting a "multi-cytokine-driven" PLR with an extremely poor outcome.

Comparison with previously published reports

Our case appears to represent a distinct entity when compared to previously published reports. While most cases of PLR in lung adenocarcinoma describe neutrophil-dominant leukocytosis, El Mouhayyar et al. reported a "triple-lineage" pattern (neutrophils, eosinophils, monocytes) without basophilia or a significant malignant effusion [9]. In our case, this ultra-aggressive phenotype, culminating in a fatal outcome within 12 days, stands in stark contrast to cases such as the anaplastic lymphoma kinase (ALK)-positive lung adenocarcinoma with PLR reported by Niu et al., where a patient survived for 5.2 months on lorlatinib [10].

While some studies suggest that tumor-associated eosinophilia does not invariably indicate a poor prognosis, as noted by Takeuchi et al. in a retrospective study of lung cancer with eosinophilic pleural effusions, the clinical context of our case is markedly different. The aforementioned study did not quantitatively analyze the proportion of eosinophils in the pleural fluid, a critical factor in our patient [11]. While a pleural fluid eosinophil fraction >10% is generally considered a significant warning sign, the proportion in most lung cancer-associated eosinophilic effusions is typically reported to be ≤15% [6,8].

In contrast, the 30.7% eosinophil fraction observed in our patient is among the highest ever reported. More importantly, this was not an isolated finding of peripheral eosinophilia. It occurred concurrently with a "quadruple-lineage" myeloid abnormality (a significant increase in neutrophils, eosinophils, basophils, and monocytes), focal eosinophilic infiltration in the pleural space, and progressive lymphocyte depletion. To our knowledge, this complex combination of findings has not been previously described in the literature and likely explains the extremely poor prognosis observed in our patient, which diverges significantly from cases characterized by isolated eosinophilia alone.

Pathophysiological hypothesis: the cytokine network

The synchronous activation of multiple myeloid lineages cannot be explained by G-CSF alone. We hypothesize that a "cytokine storm" involving tumor-derived G-CSF, granulocyte-macrophage colony-stimulating factor (GM-CSF), IL-5, and IL-6 was responsible for this presentation [12,13]. This concept aligns with the broader understanding of cytokine dysregulation in solid tumors, where excessive cytokine release can drive systemic inflammatory responses and paraneoplastic phenomena, as recently reviewed by Sun et al. [14]. In this model, GM-CSF drives the proliferation of neutrophils, eosinophils, and monocytes [7,15]; IL-5 selectively promotes eosinophil expansion and recruitment to the pleural space [9,16]; and IL-6 amplifies the systemic inflammatory response and may foster epithelial-mesenchymal transition [17,18], a finding consistent with the vimentin positivity observed in the tumor cells. The presence of bone marrow metastases suggests that the marrow microenvironment may have functioned as both a site of tumor dissemination and a pathological hub for cytokine production [19,20].

Clinical implications

A review of published PLR cases reveals a recurring pattern: bone marrow evaluation is often deferred or omitted, with diagnostic efforts focused primarily on peripheral blood findings and subsequent molecular profiling (see Appendices). This approach, however, introduces a critical delay in a clinical setting where time is paramount. While awaiting genomic results, a process that can take weeks, a patient may experience irreversible decline, as tragically occurred in this case.

Our experience underscores the value of an early bone marrow examination. This procedure, readily performed, provided a definitive diagnosis within 48 hours. Its findings were twofold and equally critical: it identified metastatic adenocarcinoma within a hyperplastic marrow, thus confirming the paraneoplastic diagnosis, while simultaneously ruling out a primary hematologic malignancy. Such rapid diagnostic clarity is fundamental to patient management, as it can avert prolonged and inappropriate antibiotic therapy and inform crucial, time-sensitive clinical decisions. Therefore, in patients presenting with unexplained hyperleukocytosis, we contend that bone marrow evaluation should be a priority investigation, pursued in parallel with molecular studies and not deferred until their completion.

## Conclusions

This report describes a case of PLR in a patient with lung adenocarcinoma, which was characterized by a distinct combination of multi-lineage granulocytic proliferation, marked eosinophilic malignant pleural effusion, and progressive lymphocyte depletion. The fulminant clinical course, which progressed over 12 days, demonstrated a highly aggressive phenotype that expands the known clinical spectrum of PLR and underscores its grave prognosis.

Clinically, these findings suggest that pronounced leukocytosis refractory to antimicrobial therapy, particularly when accompanied by eosinophilic pleural effusion and progressive lymphopenia, should prompt the strong consideration of a cytokine-driven PLR rather than an infectious etiology. Recognition of this triad may facilitate more timely diagnostic confirmation through bone marrow evaluation and prevent critical delays in management.

Furthermore, this case highlights the urgent need for mechanistic studies of multi-cytokine-driven PLR, which may identify novel therapeutic targets and create opportunities for targeted strategies, such as IL-6/signal transducer and activator of transcription 3 (STAT3) blockade.

## Appendices

**Table 2:** Detailed comparison of the present case with previously reported cases of paraneoplastic hematologic syndromes in lung cancer EPE: eosinophilic pleural effusion; PLR: paraneoplastic leukemoid reaction; NSCLC: non-small cell lung cancer; WBC: white blood cell count; G-CSF: granulocyte colony-stimulating factor; EGFR+: epidermal growth factor receptor; GM-CSF: granulocyte-macrophage colony-stimulating factor; ALK: anaplastic lymphoma kinase

Reference	Cancer type	Key hematologic features	EPE	Key distinguishing features from our case
Present case	Adenocarcinoma	Multi-lineage PLR (neutrophils, eosinophils, basophils, monocytes)+lymphopenia	Yes, marked (30.7% eosinophils)	N/A
Part A: cases of PLR, typically neutrophil-dominant				
Riesenberg et al. [21]	Adenocarcinoma	Neutrophil-dominant leukocytosis (>140×10⁹/L)	Not reported	Lacks multi-lineage complexity and significant EPE
El Mouhayyar et al. [9]	NSCLC	"Triple-lineage" (neutrophils, eosinophils, monocytes)	Not reported	Lacks basophilia, lymphopenia, and EPE
Choi et al. [2]	ALK+ adenocarcinoma	Neutrophil-dominant leukocytosis	Not reported	Neutrophil-dominant; lacks other lineage proliferation and EPE
Niu et al. [10]	ALK+ adenocarcinoma	Neutrophil-dominant leukocytosis (~179×10⁹/L)	Not reported	Neutrophil-dominant; ALK+ offers a therapeutic target
Tran et al. [4]	High-grade NSCLC	Extreme leukocytosis	Not reported	Lacks documented multi-lineage specifics and EPE
Azzam et al. [22]	Localized lung cancer	PLR, elevated WBC	Not reported	Localized disease; lacks the complexity of a hematologic picture
Yoshino et al. [23]	G-CSF-producing lung cancer	G-CSF-driven neutrophilia	Not reported	Primarily G-CSF-driven; our case suggests multi-cytokine
Part B: cases of tumor-associated eosinophilia and/or EPE				
Geng et al. [5]	EGFR+, MET amplification adenocarcinoma	Progressive isolated eosinophilia	Not reported	Isolated eosinophilia; lacks full PLR features
Kwon et al. [24]	NSCLC	Severe hypereosinophilia (>70,000/µL)	Not reported	Extreme isolated eosinophilia; lacks other PLR features
Lammel et al. [7]	Large-cell carcinoma	GM-CSF-driven eosinophilia	Yes, EPE present	Lacks multi-lineage PLR; EPE% much lower than our case
Takeuchi et al. [11]	Lung cancer cohort	EPE (≥10%)	Yes, EPE present	Cohort study; our case combines EPE with hyper-acute PLR
Lo et al. [25]	Poorly differentiated adenocarcinoma	Rapidly evolving eosinophilia	Not reported	Isolated eosinophilia; lacks EPE and other PLR lineages
